# Cross‐ validation of Free‐Cog with routinely used cognitive and functional tools in Canadian Memory Clinics

**DOI:** 10.1002/alz70858_107499

**Published:** 2025-12-25

**Authors:** Selena P Maxwell, Jodie L Penwarden, Michael Sun, Maia von Maltzahn, Shanna Claire Trenaman, Kenneth Rockwood

**Affiliations:** ^1^ Dalhousie University, Halifax, NS, Canada; ^2^ Nova Scotia Health, Halifax, NS, Canada

## Abstract

**Background:**

The Free‐Cog is a brief screening tool designed to integrate cognitive and functional measures. The Free‐Cog was previously validated against the Mini‐Addenbrooke Cognitive Examination in United Kingdom secondary care. Here, we compare the Free‐Cog with the routinely used Mini‐Mental State Examination (MMSE) and Lawton‐Brody Instrumental Activities of Daily Living (IADL) and Physical Self‐maintenance Scales (PSMS) in three Canadian Memory Clinics.

**Method:**

Records from 308 patient visits to three Memory Clinics in Nova Scotia were collected, with diagnoses available for 236 of these visits. Patients were screened with the Free‐Cog either in‐person (*n* = 278), by telephone (*n* = 17), or through video call (*n* = 12). Pearson correlation was used to examine the strength of association of Free‐Cog with MMSE, IADL, and PSMS. In‐person Free‐Cog ability to predict and discriminate dementia was determined with logistic regression and receiver operating characteristics curve (AUROC) respectively.

**Result:**

Records came predominantly from male patients (170/309, 55.0%) with dementia (149/236, 63.1%). Free‐Cog scores correlated strongly with MMSE (*r* = 0.86, 95%CI:[0.82‐0.89]; *p* <0.001), moderately with IADL (*r* = 0.56, 95%CI:[0.46‐0.64], *p* <0.001), and weakly with PSMS (*r* = 0.25, 95%CI:[0.13‐0.37], *p* <0.001). Correlations were consistent in both sexes. Telephone Free‐Cog scores only correlated with MMSE (*r* = 0.73, 95% CI: [0.39‐0.90], *p* <0.001) and virtual Free‐Cog with MMSE (*r* = 0.92, 95% CI: [0.74‐0.98], *p* <0.001) and Lawton‐Brody ADL (*r* = 0.63, 95% CI: [0.09‐0.88], *p* = 0.03). Each one‐point increase in Free‐Cog (OR=0.81, 95%CI:[0.75‐0.87], *p* <0.001), MMSE (OR=0.71, 95%CI:[0.63‐0.80], *p* <0.001), PSMS (OR=0.78, 95%CI:[0.64‐0.95], *p* = 0.01), and IADL (OR=0.60, 95%CI:[0.50‐0.73], *p* <0.001) is significantly associated with decreased odds of dementia. Stratifying by sex, Free‐Cog showed strongest association in males (OR=0.73, 95%CI:[0.65‐0.83], *p* <0.001) and MMSE in females (OR=0.81, 95%CI:[0.69‐0.95], *p* = 0.008). Free‐Cog (AUROC=0.86) demonstrated the best ability at discrimination of dementia cases followed by MMSE (0.73), IADL (0.69), whereas PSMS (0.59) could not. MMSE was best at discriminating dementia expression in males (0.79) and females (0.73).

**Conclusion:**

The Free‐Cog test appears to be a valid alternative to the routinely used MMSE and Lawton‐Brody scales for dementia screening. A more robust sample is required to determine the effectiveness of the telephone and virtual versions of Free‐Cog as well as test‐specific sex differences.

